# Contribution of telomerase RNA retrotranscription to DNA double-strand break repair during mammalian genome evolution

**DOI:** 10.1186/gb-2007-8-12-r260

**Published:** 2007-12-07

**Authors:** Solomon G Nergadze, Marco Andrea Santagostino, Alberto Salzano, Chiara Mondello, Elena Giulotto

**Affiliations:** 1 Dipartimento di Genetica e Microbiologia 'Adriano Buzzati-Traverso', Università degli Studi di Pavia, Via Ferrata, 27100 Pavia, Italy; 2 Istituto di Genetica Molecolare, CNR, Via Abbiategrasso, 27100 Pavia, Italy

## Abstract

**Background:**

In vertebrates, tandem arrays of TTAGGG hexamers are present at both telomeres and intrachromosomal sites (interstitial telomeric sequences (ITSs)). We previously showed that, in primates, ITSs were inserted during the repair of DNA double-strand breaks and proposed that they could arise from either the capture of telomeric fragments or the action of telomerase.

**Results:**

An extensive comparative analysis of two primate (*Homo sapiens *and *Pan troglodytes*) and two rodent (*Mus musculus *and *Rattus norvegicus*) genomes allowed us to describe organization and insertion mechanisms of all the informative ITSs present in the four species. Two novel observations support the hypothesis of telomerase involvement in ITS insertion: in a highly significant fraction of informative loci, the ITSs were introduced at break sites where a few nucleotides homologous to the telomeric hexamer were exposed; in the rodent genomes, complex ITS loci are present in which a retrotranscribed fragment of the telomerase RNA, far away from the canonical template, was inserted together with the telomeric repeats. Moreover, mutational analysis of the TTAGGG arrays in the different species suggests that they were inserted as exact telomeric hexamers, further supporting the participation of telomerase in ITS formation.

**Conclusion:**

These results strongly suggest that telomerase was utilized, in some instances, for the repair of DNA double-strand breaks occurring in the genomes of rodents and primates during evolution. The presence, in the rodent genomes, of sequences retrotranscribed from the telomerase RNA strengthens the hypothesis of the origin of telomerase from an ancient retrotransposon.

## Abstract 2

A comparative analysis of two primate and two rodent genomes suggests that telomerase was utilized, in some instances, for the repair of DNA double-strand breaks during mammalian evolution.

## Background

The vertebrate telomeres consist of extended arrays of the TTAGGG hexamer. The specialized function of the telomerase enzyme, together with a multitude of telomere-binding proteins, is required to maintain sufficiently long telomeres, assuring stability to the linear eukaryotic chromosomes. Telomerase is an atypical reverse transcriptase that adds telomeric repeats to chromosome ends, overcoming the limitations of the replicative apparatus that would cause shortening of the termini at each replication round. Telomerase is composed of two moieties: a protein endowed with reverse transcriptase activity (telomerase reverse transcriptase (TERT)), and an RNA molecule (telomerase RNA component (TERC)) [1-3]. Telomerase utilizes a portion of its RNA component as a template for the synthesis of telomeric repeats. The structure of the telomerase RNA component has been studied in several organisms; its size ranges between 382 and 559 nucleotides [4,5] in vertebrates, whereas it is significantly larger in yeast (of the order of 1,000 nucleotides or more) [6] and shorter in ciliates (146-205 nucleotides) [7,8]. The vertebrate TERCs possess a conserved secondary structure: a pseudoknot at the template-containing 5' end, and three partial stem-loop arms. The mouse and human TERCs have a very similar sequence and structure except for their 5' ends: in humans the telomeric repeat template lies 45 nucleotides away from the 5' end, whereas in mouse, as well as in other rodents (rat and Chinese hamster), it is only two nucleotides removed [4,9,10].

Repetitions of the telomeric hexamer at intrachromosomal sites, the so called interstitial telomeric sequences (ITSs), have been described in many species, including primates and rodents [11-16]. In previous work [17], we cloned 11 ITS loci from 12 primate species and demonstrated that they were introduced during the repair of DNA double-strand breaks that were fixed in the genome in the course of evolution. The telomeric repeat insertion occurred either without modification of the sequence at the break site or with processing of the ends produced by the break involving deletions, insertions or target site duplications [17] (Additional data file 1). These observations are in agreement with the results obtained by several authors showing that the standard repair of double-strand breaks via non-homologous end-joining occurs together with modifications of the break site [18-22]. We then proposed that the addition of telomeric repeats at the break site could be due to either the action of telomerase or the capture of telomeric fragments, as shown in Additional data file 1.

A direct involvement of telomerase in ITS insertion is conceivable in view of the mounting evidence for the sharing of factors between the machineries for DNA double-strand break repair and telomere maintenance [23-27]. In particular, many DNA repair proteins, such as the DNA-end binding Ku heterodimer, the catalytic subunit of the DNA dependent protein kinase, the ERCC1/XPC and Werner helicases, and the Mre11/Rad50/Nbs complex, interact also with telomeres [28-32]. Reciprocally, the telomeric repeat factor 2 protein (TRF2) can be recruited at DNA double-strand breaks [33].

In order to investigate the possible role of telomerase in ITS insertion, we took advantage of the availability of the nearly complete sequence of the genomes of *Homo sapiens*, *Pan troglodytes*, *Mus musculus *and *Rattus norvegicus *to analyze all the ITSs present in them. We were thus able to demonstrate that the same mechanisms for ITS insertion, previously identified in primates, are also operating in rodents. Furthermore, we obtained evidence that, in rodents, portions of TERC other than the canonical hexameric template can be retrotranscribed during the process; this observation, together with the results obtained by a comparative analysis of all ITS loci, suggests that telomerase can contribute to DNA double-strand break repair.

## Results

### Search of rodent and primate ITSs

Using the (TTAGGG)_4 _sequence as query, we performed a BLAT search [34,35] for all the interstitial telomeric loci present in the genome sequence of two species of the Rodentia order, muridae family (*M. musculus *or mouse and *R. norvegicus *or rat) and two species of the Primates order, hominidae family (*H. sapiens *or human and *P. troglodytes *or chimpanzee). We found 306 and 326 ITS loci in the mouse and rat genomes, respectively, and 100 and 110 ITS loci in the human and chimpanzee genomes, respectively, containing four or more TTAGGG repeated units. Subtelomeric type loci consisting of tandemly oriented exact and degenerate TTAGGG repeats were preliminarily removed since they are probably the product of recombination events involving telomeres [36]. This operation left 244 mouse, 250 rat, 83 human and 79 chimpanzee ITSs with at least four TTAGGG units and less than one mismatch per unit. A complete list and description of the ITS loci used for this analysis is presented in the Additional data files 2-8.

### Search of species-specific ITS and mechanisms of ITS insertion: rodent-primate comparison

For each mouse ITS locus, we searched the orthologous rat locus by using up to 20 kb of the sequence comprising the ITS as query for a BLAT search against the rat genome database. Similarly, the mouse loci orthologous to rat ITS loci were searched in the mouse genome database. For 128 mouse and 120 rat loci the orthologous loci in the other species were either not identifiable or grossly rearranged (Tables S1 and S2 in Additional data file 2). In 58 loci the telomeric repeats were conserved in both species (Table S3 in Additional data file 3), hence they were inserted in the genome of a common ancestor of mouse and rat (more than 12-14 million years ago (MYA)) [37]. Finally, for 58 mouse and 72 rat ITSs the orthologous loci in the other species were clearly identified and did not contain the telomeric-like repeats (Tables S4 and S5 in Additional data file 4). These ITSs were called 'species-specific' since they were inserted after the mouse/rat split, that is, less than 12-14 MYA.

The same type of comparative analysis was carried out for the 83 human and the 79 chimpanzee ITSs. The majority (75 loci) of the primate ITSs (83 total human loci and 79 total chimpanzee loci) were present in both species (Additional data file 5), hence they originated before the human/chimpanzee split, that is, more than 6 MYA [38]. Only for three human ITSs were the orthologous chimpanzee loci highly rearranged (Tables S6 and S7 in Additional data file 5). Therefore, only five human-specific and four chimpanzee-specific ITSs could be found (Table S8 in Additional data file 6).

By comparing the flanking sequence of each ITS-containing locus with the sequence of the corresponding empty locus in the two Rodentia and the two Primates species, we could define the mechanism of insertion at each informative locus (examples of the sequences used for this analysis are shown in Additional data file 7). We found that the ITSs were inserted with the same mechanisms previously described in primates [17], which thus also operate in rodents. Interestingly, the frequency of the different mechanisms was also similar in the two orders (Table 1).

**Table 1:** Mechanisms of ITS insertion

	Number of loci						
	
	Rodents			Primates			
		
Flanking sequence modification	Mouse	Rat	Total (%)	Human	Chimp	Both*	Total (%)
No modification	15	16	31 (23,2)	1	0	3	4 (16)
Deletion	27	38	65 (50)	2	2	9	13 (52)
Addition	10	12	22 (17)	1	1	2	4 (16)
Random sequence	8	12					
TERC sequence^†^	2	0					
Duplication	2	4	6 (4,6)	1	1	2	4 (16)
Addition and deletion	4	2	6 (4,6)	0	0	0	0 (0)
Random sequence addition	1	2					
TERC sequence addition^†^	3	0					
Total	58	72	130 (100)	5	4	16	25 (100)

*These ITSs are present in both primate species and were inserted within repetitive elements. Their insertion mechanism was defined from the repetitive element consensus. ^†^TERC sequence additions are present only in rodents.

Surprisingly, at some rodent loci, the ITS was added together with a sequence homologous to a portion of a TERC distant from the telomeric template. These loci and the proposed mechanism of insertion are discussed below.

### Length and telomeric sequence conservation of rodent and primate ITSs

**Figure 1:**
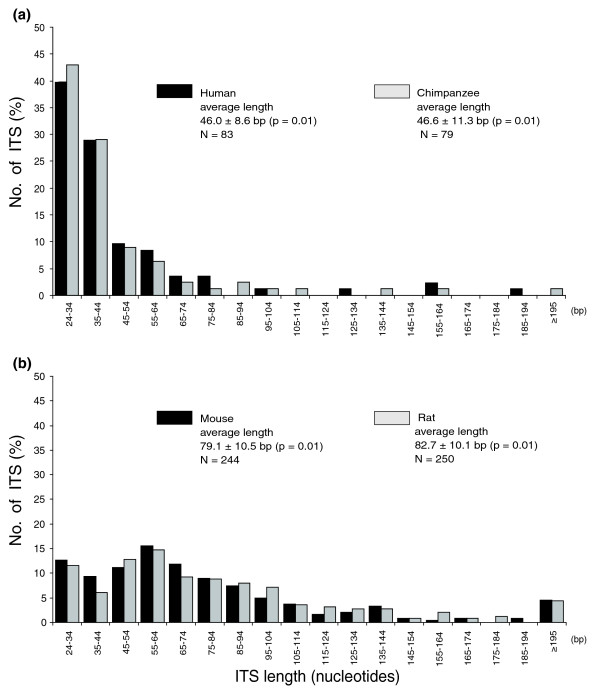
Length of ITSs. Comparison of ITS length in **(a) **the two primate and **(b) **the two rodent species.

The analysis of the length of all the interstitial telomeric arrays (reported in Tables S1-S8 in Additional data files 2-6) has shown that the length of the ITSs is similar in mice as compared to rats and in humans as compared to chimpanzees (Figure 1). However, on average, the rodent ITSs are significantly longer than the primate ones: the majority of the primate ITSs (71% in humans and 75% in chimpanzees) are shorter than 50 bp whereas 70% of mouse and 73% of rat ITSs are longer than 50 bp. The ITS length reported here refers to the sequences from the database, whereas length polymorphism was observed in different mouse individuals (unpublished observation), similar to what we have previously shown in humans [39].

An overall comparison of the ITSs found in the four species is reported in Tables 2 and 3. The proportion of primate ITSs conserved in both species is very high (more than 90% in both humans and chimpanzees), and significantly higher than in rodents (close to 24% in both mice and rats). As mentioned above, the conserved ITSs were inserted more than 6 MYA in the primate genome and more than 12-14 MYA in the rodent genome. Conversely, the proportion of species-specific, that is, relatively 'young' ITSs, is much higher in the rodent (approximately one out of four) than in the primate species (approximately one out of 20). The species-specific ITSs were inserted in the primate and rodent genomes less than 6 MYA and less than 12-14 MYA, respectively. A much higher proportion of loci for which the orthologous ones could not be found or were highly rearranged was also observed in rodents compared to primates (not informative loci in Table 2, listed in Tables S1, S2 and S7 in Additional data files 2 and 5).

**Table 2:** ITS age

	Number of ITS (%)			
	
ITS type	Human	Chimpanzee	Mouse	Rat
Conserved ITS loci (old)	75 (90.4)	75 (94.9)	58 (23.8)	58 (23.2)
Species-specific ITS loci (young)	5 (6.0)	4 (5.1)	58 (23.8)	72 (28.8)
Not informative ITS loci*	3 (3.6)	0 (0)	128 (52.5)	120 (48.0)
Total	83	79	244	250

*ITS loci for which the orthologous loci were not found or were grossly rearranged.

**Table 3:** Telomeric sequence mutation

	Number of mismatches per TTAGGG unit			
	
ITS type	Human	Chimpanzee	Mouse	Rat
Conserved ITS loci (old)	0.29 ± 0.07	0.30 ± 0.08	0.40 ± 0.13	0.34 ± 0.09
Species specific ITS loci (young)	0.13 ± 0.12	0	0.14 ± 0.03	0.12 ± 0.03

Since, in several ITSs, nucleotides diverging from the canonical telomeric hexamer (mismatches) were observed (Tables S1-S8 in the Additional data files 2-6), we wondered whether their frequency was correlated with the age of the insertion event. Considering that the species-specific ITSs were inserted in the genome more recently than the conserved ones, we compared the frequency of mismatches in species-specific and in conserved ITSs. In all four species, the number of mismatches per telomeric unit is significantly lower in the 'young' (species-specific) compared to the 'old' (conserved) ITSs (Table 3); therefore, the 'old' conserved ITSs accumulated more mutations.

### Microhomology between break sites and inserted telomeric repeats

If telomerase was directly involved in the insertion of ITSs at break sites, we would expect, in the ancestral sequence, a non-random presence of nucleotides in register with the inserted telomeric repeats. In fact, the presence of 1-5 nt microhomology to the telomeric hexamer at the 3' end of a break site is known to favor so called 'chromosome healing', that is, the creation of a new telomere at a break site by telomerase [40,41]. We therefore analyzed the species-specific ITSs by comparing their flanking sequences with the ancestral empty sequences in order to determine whether the 3' end of the break, in the ancestral sequence, exposed nucleotides in register with the inserted telomeric repeats.

**Figure 2:**
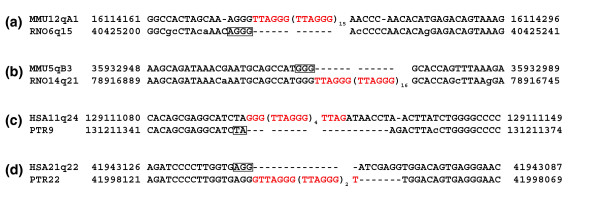
Microhomology between break sites and inserted telomeric repeats. Telomeric repeats are in red; in the empty ancestral loci the nucleotides in register with the inserted telomeric repeats are boxed. **(a) **Mouse specific ITS at the MMU12qA1 locus; an AGGG tetranucleotide from the orthologous rat empty locus RNO6q15 is in register with the inserted telomeric repeats. **(b) **Rat specific ITS at RNO14q21; a GGG trinucleotide from the orthologous mouse locus MMU3qB3 is in register with the inserted telomeric repeats. **(c) **The human specific ITS at HSA11q24 was inserted together with seven random nucleotides; a TA dinucleotide from the orthologous chimpanzee PTR9 locus is in register with the inserted telomeric repeats.**(d) **The insertion of the chimpanzee specific ITS at PTR22 occurred together with a 7 bp deletion; an AGG trinucleotide from the orthologous human locus HSA21q22 is in register with the inserted telomeric repeats.

The results of this analysis showed a strikingly high frequency of nucleotides in register with the inserted telomeric repeats (see Tables S4, S5 and S8 in Additional data files 4 and 6 for a complete list, Figure 2 for some examples and Table 4 for a quantitative analysis).

**Table 4:** Number of loci containing nucleotides in register with the telomeric insertion*

	Number of observed loci (%)				
		
No. of nucleotides in register with telomeric insertion	Mouse	Rat	Human	Chimpanzee	No. of expected loci (%)
0^†^	11 (23)	16 (25)	1 (20)	1 (33)	(75)
1 or more^‡^	36 (77)	47 (75)	4 (80)	2 (67)	(≤ 25)
2 or more^§^	26 (55)	31 (49)	3 (60)	2 (67)	(≤ 6.25)
3 or more^¶^	16 (34)	21 (33)	2 (40)	1 (33)	(≤ 1.56)
4 or more^¥^	8 (17)	11 (17)	1 (20)	0 (0)	(≤ 0.39)

* For this analysis we utilized the informative species-specific loci listed in Additional data files 4 and 6, namely 47 mouse, 63 rat, 5 human and 3 chimpanzee ITS loci. ^†^This class includes the loci in which the last nucleotide at the 3' end of the break is not in register with the inserted telomeric repeat insertion. ^‡^This class includes all loci with in register nucleotides. This class, together with the previous class comprises the totality of the loci. ^§^These loci are also included in the '1 or more' class. ^¶^These loci are also included in the '1 or more and 2 or more' classes. ^¥^These loci are also included in the '1 or more, 2 or more and 3 or more' classes.

In Table 4 the frequency of loci with microhomology with the inserted telomeric sequence at the break site is shown. For this analysis we utilized the informative species-specific loci listed in Tables S4, S5 and S8 in Additional data files 4 and 6, namely 47 mouse, 63 rat, 5 human and 3 chimpanzee ITS loci. If the addition of TTAGGG repeats did not involve telomerase, we would expect that the ancestral loci lacking the repeats would contain random nucleotides at the break site. In this hypothesis, nucleotides homologous to the inserted telomeric repeats would be due to chance; therefore, the expected percentage of loci in which the last nucleotide at the break site is not in register would be 75% whereas the observed percentage of such loci is only around 25% in all species. Conversely, the frequency of loci bearing microhomology with the telomeric insertion at the break site is much higher than expected from randomness; in fact, one or more (up to eight) homologous nucleotides were observed in 77% of the mouse, 75% of the rat, 80% of the human and 67% of the chimpanzee informative loci while their expected frequency is less than 25%. The difference between expected and observed frequencies is even more striking if we consider the loci with more than one nucleotide in register: for example, the expected frequency of insertions with homology of three or more nucleotides arising from random events would be less than 2% whereas we observed at least 33% frequency for such loci in all species. These observations strongly suggest the involvement of telomerase in the process.

### Search for TERC-ITS loci

The analysis of the sequences flanking the telomeric repeats produced a surprising result: in the mouse and rat genomes ITSs were sometimes adjacent to a sequence identical to the 3' domain of the RNA component of telomerase. Following this observation, we carried out a thorough search for ITS loci containing non-telomeric TERC sequences (TERC-ITS loci). An exhaustive BLAT search of loci containing TERC-like sequences was performed in the genome of the four species using the TERC genes as query. In the primate genomes no homologies were scored besides the TERC gene itself. On the contrary, in the mouse, 14 loci containing portions of the TERC sequence different from the repeat template were found adjacent to telomeric repeats (Table 5). Three loci (1 to 3 in Table 5) are conserved in mouse and rat; nine loci (4-12 in Table 5) are present only in the mouse and the rat orthologous loci, lacking TERC-like and ITS inserts, were identified; for two additional mouse loci the orthologous rat locus could not be found (13 and 14 in Table 5). Finally, a TERC pseudogene is included in a duplicon, located on chromosome 3 (MMU3qA3 nt 30005830, data not shown), 65 Mb away from the TERC gene itself. In the rat genome, besides the three loci that are conserved in the mouse (1, 2 and 3 in Table 5), two rat specific loci containing TERC-like sequences were found (RNO2q21 nt 70846447 and RNO4q42 nt 154642330, data not shown); one of these contains a 74 bp uninterrupted fragment homologous to nucleotides 322-395 of the TERC RNA; the other one contains a 117 bp uninterrupted fragment homologous to nucleotides 3-119 of the telomerase RNA. These two rat loci are not discussed here since they do not comprise TTAGGG repeats and, therefore, can be considered short pseudogenes that did not necessarily derive from the mechanisms under study.

**Table 5:** Mouse loci containing TERC-like sequences

	Mouse locus organization					Orthologous rat locus organization		
		
	Chromosomal localization	Starting nucleotide of fragment homologous to 3' TERC domain (length)	Position within TERC sequence	No. of nucleotides complementary to sequence preceding template	ITS length	Chromosomal localization	Starting nucleotide of TERC fragment (length)	Starting nucleotide of ITS (length)
1	MMU8qA2	21522357 (38)	351-388	0	213	RNO16q12	73726184 (38)	73726146 (58)
2	MMU13qA1	3475939 (54)	331-384	0	22	RNO17q12	77825660 (53)	77825608 (22)
3	MMUXqC3	94682056 (52)	328-377	7	25	RNOXq31	88164322 (52)	88164265 (43)
4	MMU5qA3	23908490 (37)	357-393	6	21	RNO3q41	139145891	No TERC, no ITS
5	MMU9qA5	47975305 (60)	314-373	6	68	RNO8q23	51245798	No TERC, no ITS
6	MMU1qC1	47024551 (31)	341-374	3	57	RNO9q22	52312295	No TERC, no ITS
7	MMU4qD2	119006678 (42)	351-392	6	53	RNO5q36	140562692	No TERC, no ITS
8	MMU10qB4	58505103 (118)	271-388	0	27	RNO20q11	37230663	No TERC, no ITS
9	MMU12qF1	106800391 (81)	308-388	0	13	RNO6q32	136092775	No TERC, no ITS
10	MMUXqA6	61359852 (74)	322-395	6	23	RNOXq37	152798431	No TERC, no ITS
11	MMU1qC3	69326421 (50)	346-395	6	139	RNO9q32	68032617	No TERC, no ITS
12	MMU10qA3	20387038 (98)	289-388	6	108	RNO1p12	15780743	No TERC, no ITS
13	MMU6qC1	68259720 (44)	351-394	0	93	Not found	-	-
14	MMU11qC	86742217 (38)	343-381	0	55	Not found	-	-

### Organization of TERC-ITS loci

**Figure 3:**
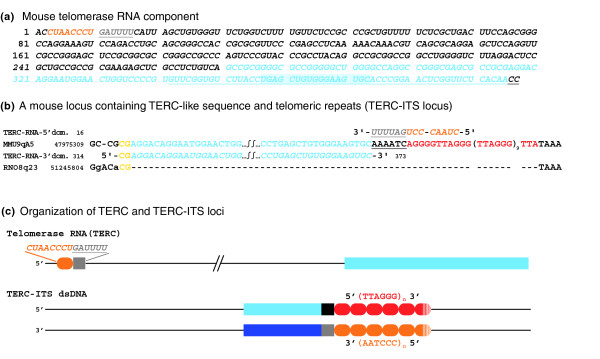
Organization of TERC-ITS loci. RNA sequences are in italic. The RNA sequences involved in the events and the DNA sequences corresponding to them (that is, complementary to retrotranscribed sequences) are in light colors (orange, grey and light blue) while the DNA sequences derived from retrotranscription of the RNA are in dark colors (red, black and dark blue). **(a) **Sequence of the mouse telomerase RNA component. The nucleotides of the canonical telomerase template, located near the 5' end, are shown in orange (nucleotides 3-10). Nucleotides adjacent to the template that are retrotranscribed together with the first inserted hexamer are grey underlined. The nucleotides of the 3' domain of TERC involved in the TERC-ITS loci are indicated in light blue. The 17 nt core sequence, present in all TERC-ITSs, has a blue background. In the 3' domain of the RNA, the mouse TERC sequence homologous to the human TERC sequence interacting with Ku is underlined. **(b) **Example of a mouse specific TERC-ITS locus (MMU9qA5). The top row shows the 5' domain of TERC containing the canonical template (orange) and the adjacent sequence (grey underlined). The second row shows the sequence of the mouse locus: telomeric repeats are in red; the nucleotides complementary to those adjacent to the hexameric template are black underlined; the light blue nucleotides indicate the region derived from the 3' domain of TERC. The third row reports, in light blue, the sequence of the 3' domain of TERC from nucleotides 314 to nucleotides 373. The bottom row shows the sequence of the orthologous empty rat locus RNO8q23. The CG dinucleotide (yellow) is present both in the ancestral rat sequence, at the 3' end of the break, and in the region of the TERC RNA immediately preceding the retrotranscribed 3' domain. **(c) **Overall organization of TERC-ITS loci. At the top is the structure of TERC: orange oval, canonical template; grey square, adjacent nucleotides; light blue strip, 3' domain. At the bottom is the organization of the double-stranded DNA at TERC-ITS loci: light blue strip, sequence corresponding to the 3' domain of TERC; blue strip, complementary sequence; black square, sequence complementary to the nucleotides adjacent to the canonical template; grey square, sequence corresponding to the nucleotides adjacent to the canonical template; red ovals, TTAGGG repeats; orange ovals, complementary repeats.

Figure 3 reports the sequence of mouse TERC (Figure 3a), the sequence of a mouse-specific TERC-ITS locus (Figure 3b) and a sketch of the organization of TERC-ITS loci (Figure 3c). In Figure 3a the canonical telomerase template, located near the 5' end, is shown in orange (nt 3-10). All the 14 loci listed in Table 5 contain, besides a repetition of the telomeric hexamer, a sequence homologous to the 3' domain of the RNA, varying in length between 31 and 118 nt (Table 5) but always comprising between nucleotides 271 and 395 of the 397 nt-long mouse TERC (light blue nucleotides in Figure 3a). A 17 nt core sequence (blue background in Figure 3a) is always present. In Figure 3a the mouse TERC sequence homologous to the human TERC sequence interacting with Ku [42] is underlined (nucleotides 342-397); it is worth mentioning that the core sequence is contained within the postulated Ku-interacting region. All insertions of the 3' domain of TERC are followed by variable numbers of TTAGGG repeats. One example is shown in Figure 3b, in which the insertion of TERC related sequences occurred in a mouse ancestor after its divergence from the rat lineage. The mouse sequence (MMU9qA5) contains a 60 nt fragment homologous to the 3' portion of TERC; at this locus, as in seven other loci (see Additional data file 8), the telomeric repeats are preceded by a few nucleotides complementary to the sequence immediately preceding the 3' side of the canonical template (grey underlined nucleotides in Figure 3a). Surprisingly, the fragments corresponding to the 3' domain of TERC and those corresponding to the telomeric repeats (derived from the 5' domain of TERC) are in opposite orientation to each other. In other words, whereas the 5' domain is retrotranscribed from the template RNA, the 3' domain is complementary to a retrotranscribed sequence. A CG dinucleotide (yellow in Figure 3b) is present both in the ancestral rat sequence, at the 3' end of the break, and in the region of the telomerase RNA immediately preceding the retrotranscribed 3' domain. This microhomology could help in positioning the RNA before retrotrascription. For a complete description of the organization of all 14 mouse loci containing insertions of the 3' moiety of TERC, see Additional data file 8. The overall organization of these loci is schematized in Figure 3c.

## Discussion

### Comparison of rodent and primate ITSs

In our previous work [17] we described the mechanisms for insertion of telomeric repeats in primate genomes during the repair of DNA double-strand breaks. Here, we confirm these mechanisms in primates and find that they are operational also in rodents. Primate and rodent ITSs, unlike other microsatellites, appeared in one step during evolution, inserted in a pre-existing and well conserved unrelated sequence. This feature indicates that the ITSs described here are not generated by telomeric fusion. The birth of ITSs is based on mechanisms clearly distinct from the mechanism of origin of classical microsatellites, that is, the creation of a minimum number of repeat units by mutation followed by repeat expansion through DNA polymerase slippage [43]. Table 1 shows that the frequency of the different insertion mechanisms is similar in the two mammalian orders, the insertion events involving deletions of flanking sequences being the most represented both in rodents and in primates. Deletions of broken ends before joining were indeed the most frequent modification observed in several experimental systems in which the junctions produced after the repair of enzymatically induced breaks were sequenced [18-22]. The data presented do not allow us to estimate the probability of ITS insertion in mammalian genomes. However, considering that we observed 244, 250, 83 and 79 ITSs in the mouse, rat, human and chimpanzee genomes, respectively, and that many others should have occurred without being fixed during evolution, we can conclude that the frequency of this event is not negligible. However, ITS insertion was never detected at experimentally induced DNA double-strand breaks in both human and rodent cultured somatic cells [22]; thus, either this type of event cannot occur in somatic cells or its frequency is too low to be detected in the experimental systems used.

It has been suggested that the presence of telomeric-like repeats at interstitial sites may cause chromosomal instability [44-47]; in light of the results of our work, we suggest the alternative hypothesis that ITSs themselves are not fragile sites but were inserted within fragile sites and can, therefore, be considered relics of ancient breakage.

Although the four basic mechanisms of ITS insertion are shared between primates and rodents, the presence, at 14 mouse ITS loci, of sequences homologous to the 3' domain of TERC revealed that, in rodents, an additional mechanism, involving TERC retrotranscription, was active. This pathway is present only in the rodents and is discussed below.

Another difference between the two orders is the length of the ITSs (Figure 1): about 46 nucleotides, on average, in primates and about 81 nucleotides in rodents. This difference may derive from properties of the rodent and primate telomerases. It is well known in fact that the telomeres themselves are much longer in rodents (up to 150 kb) [48] than in primates (up to 25 kb) [49,50], in spite of the fact that the human telomerase seems to be more processive than the mouse enzyme [51].

The proportion of primate ITSs conserved in both species, and therefore inserted before the human-chimpanzee split, is very high (more than 90%), and significantly higher than in rodents (24%) (Table 2). Conversely, the proportion of species-specific ITSs, that is, inserted after either the human-chimpanzee split or the mouse-rat split, is much higher in rodents compared to primates. This is in agreement with the fact that the two primate species separated more recently (6 MYA) [38] than the two rodent species (12-14 MYA) [37] and underwent fewer generations per unit time. Even more relevant to this regard could be the high rate of mutation and rearrangement [52,53] of the rodent genomes with respect to those of other mammals. The same reasons can explain the much higher proportion of rodent loci for which the orthologous ones could not be found or were highly rearranged (not informative loci in Table 2, listed in Tables S1, S2 and S7 in Additional data files 2 and 5).

In all four species, the number of mismatches per telomeric unit is significantly lower in the 'young' (species-specific) compared to the 'old' (conserved) ITSs (Table 3): the 'old' conserved ITSs accumulated more mutations. This observation is consistent with the hypothesis that ITSs were inserted in the genomes as exact arrays of the telomeric unit, which then accumulated mutations in the course of evolution.

### Role of telomerase in ITS production

In our previous work, we proposed that the ITSs could be inserted at DNA double-strand break sites either by telomerase or by the capture of telomeric fragments [17]. The results presented here support the hypothesis that telomerase is directly involved in the process, although its intervention in double strand break repair is probably a rare event and its consequence can be observed only on an evolutionary time scale. Participation of telomerase to ordinary double strand break repair might not be a general mechanism because it would produce the insertion of telomeric repeats during end-joining but also extensive chromosome fragmentation through chromosome healing. To this regard, it is worth mentioning that in a yeast experimental system, in which sequence-specific double-strand breaks were induced in strains defective in homologous recombination, telomerase was recruited at double-strand breaks approximately 1% of the time, giving rise to new telomeres (chromosome healing) [54].

Two independent sets of data presented in this work point to a direct role of telomerase in ITS formation. In the first place, in a highly significant number of species-specific loci, the break site, which occurred in the ancestral sequence, exposed from one to eight nucleotides in register with the inserted telomeric hexamers. Even more significant in this regard is the observation that, at 14 mouse ITS loci, sequences homologous to the 3' domain of the RNA component of telomerase, far away from the hexamer template, which is located near the 5' end of the RNA, were inserted together with the telomeric repeats (Figure 3, Table 5 and Additional data file 8).

All these loci share a peculiar organization of the TERC related sequences (Figure 3c): the telomeric repeats are preceded by a 31-118 nt fragment homologous to a portion of the 3' domain of TERC (comprising nucleotides 271-395 and always containing a 17 nucleotide core sequence; Figure 3a) and the 5' and 3' domains of TERC are inserted in opposite orientations. Furthermore, in 8 of the 14 loci the telomeric repeats are preceded by a few nucleotides complementary to the sequence immediately preceding the 3' side of the canonical template (Table 5, Additional data file 8, and black or grey underlined nucleotides in Figure 3). Finally, in seven out of the eight informative examples, microhomology is observed between the 3' end of the break in the ancestral sequence and the nucleotides immediately preceding the retrotranscribed TERC 3' domain (yellow nucleotides in Figure 3b and in Additional data file 8). These findings clearly point to the involvement of telomerase in the insertion process. This inference is justified by the increasing body of data showing that several proteins involved in the repair of those breaks are also involved in telomere maintenance [23-33]. Yet, this hypothesis implies a relatively complex model to justify two puzzling observations: the inverted orientation of the 3' domain-derived fragment with respect to the telomeric repeats; and the presence, in most cases, of a few nucleotides complementary to the sequence preceding the hexameric template. Several models have been proposed to explain endonuclease-independent retrotrasposition events [55-58]. None of these models can justify the insertion of sequences with opposite orientation from the same template RNA. An elegant model has been proposed by Ostertag and Kazazian [59] to explain the creation of inversions in L1 retrotrasposition. This model is a modification of target primed reverse transcription involving twin priming. In this process retrotranscription of the two regions of the RNA is primed by the 3' ends of the two sides of the break. However, this model cannot explain the organization of the TERC-ITSs we have observed. In fact, it would produce a sequence in which the telomeric repeats would be primed by one end of the break towards the center of the break and the nucleotides immediately preceding the canonical template would be added directly at the break site. In our case instead, the nucleotides preceding the telomeric repeats (black underlined in Figure 3b) are located in the center of the insertion and not at the break site and are followed by telomeric repeats (red in Figure 3b) in the same orientation. Therefore, a different mechanism must operate in the process described here.

### A model for the mechanism of TERC-like fragment insertion

**Figure 4:**
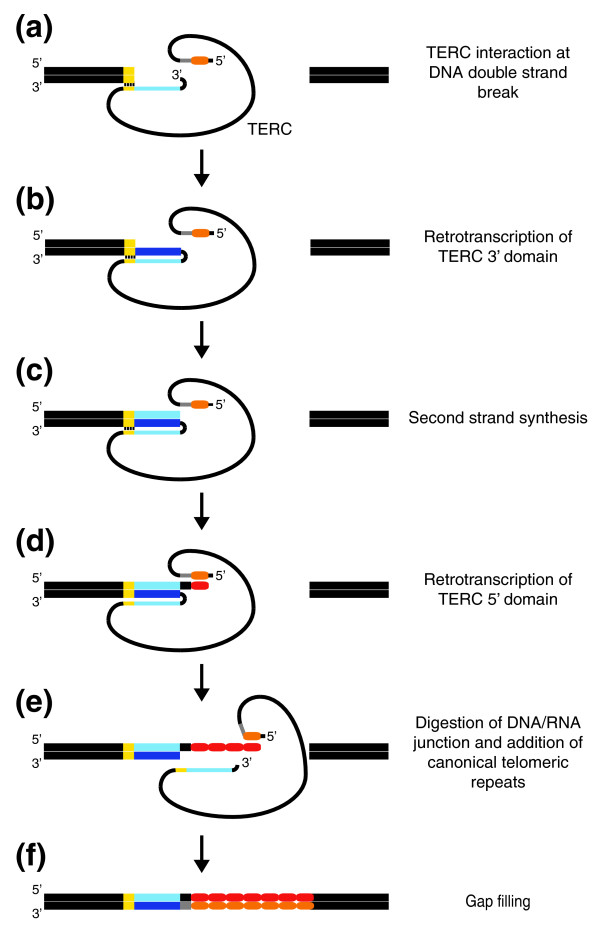
Model for TERC-ITS insertion. **(a) **TERC interaction at DNA double strand break. **(b) **Retrotranscription of TERC 3' domain. **(c) **Second strand synthesis. **(d) **Retrotranscription of TERC 5' domain. **(e) **Digestion of DNA/RNA junction and addition of canonical telomeric repeats. **(f) **Gap filling. Curved thin lines represent the telomerase RNA (TERC) in which the orange oval corresponds to the canonical telomeric template, the grey line to the nucleotides immediately adjacent to the 3' side of the template, the yellow line to nucleotides homologous to the last nucleotides of the break site; the light blue line represents the retrotranscribed 3' region of TERC. Straight thick lines represent DNA strands. The DNA involved in the double-strand break is in black, the yellow boxes correspond to nucleotides homologous to the region of TERC preceding the sequence retrotranscribed from the 3' end, the dark blue line represents the DNA strand retrotranscribed from the 3' end of TERC and the light blue line is the complementary strand. Red and orange ovals represent TTAGGG and CCCTAA repeats, respectively. Black and grey lines correspond to the sequence homologous to the nucleotides immediately adjacent to the telomeric template.

Figure 4 shows a possible model to explain the structural oddities of the observed TERC-ITSs. In the first place, we assume that the two DNA ends derived from a double-strand break are maintained in contact (Figure 4a), possibly by the interaction with Ku, which has a specific affinity for double-strand ends. Ku also has a specific affinity for the 3' portion of TERC [5,42,60], which could thus conceivably be brought into close contact with a broken end (Figure 4a), as well as an affinity for TERT [42,60], which, of course, in its turn, tends to bind TERC and DNA ends. We then propose that the 3' end of the RNA can fold back to act as a primer for retrotranscribing into DNA a portion of its 3' sequence until it reaches the 5' end of the DNA break (Figure 4b); this reaction could be favored by microhomology between the last nucleotides at the break and the RNA (short vertical bars in Figure 4a-c), thus helping the RNA/DNA alignment. In fact, in seven out of the eight loci that are informative to this regard, an identical stretch of one to five nucleotides is present in the ancestral sequence, at the break site, and in the region of the telomerase RNA immediately preceding the retrotranscribed fragment (yellow nucleotides in Figure 3b and Additional data file 8). The retrotrascription could be performed by a TERT molecule bound to TERC or by another reverse transcriptase. At this point, the 3' end of the break could offer a primer for a DNA-dependent DNA polymerase to copy the retrotranscribed stretch (Figure 4c). Now, we assume that the canonical template is brought into contact with the newly polymerized 3' end. Thus, the first telomeric monomer can be added by retrotranscription together, in most cases, with a few nucleotides complementary to those on the 3' side of the template (Figure 4d). This step provides a seeding sequence for telomerase to act in its standard way, adding a certain number of hexamers (Figure 4e). Finally, a filling by DNA polymerase and a ligation step complete the reconstitution of duplex integrity (Figure 4f).

It is conceivable that several non-homologous end joining (NHEJ) proteins may play a role in different steps of this process, as well as in the simple insertion of telomeric repeats. In particular, besides Ku, which is known to bind the telomerase RNA component, the DNA-PK catalytic subunit may be involved in the activation of factors responsible for the final end-joining. In addition, the observation that sequences at the break site are modified during ITS insertion (Table 1 and Additional data file 7) suggests that NHEJ nucleases such as Artemis are involved in the processing of DNA ends [61]. It has been proposed that double strand break proteins, including Ku, can temporarily allow access of telomerase to internal double-strand breaks, promoting the formation of a new telomere [27]. During the formation of ITS or TERC-ITS loci, telomerase is recruited to double-strand breaks, but only a limited number of telomeric repeats is synthesized and the integrity of the original chromosome is restored.

The model presented in Figure 4 has the advantage of explaining, in an economic way, the peculiarities of orientation and sequence composition of the inserts and is consistent with the known properties of the factors involved, including the observation that Ku is also involved in telomere maintenance. In addition, the model could justify the fact that, in spite of the overall similarity of the mouse and human TERC structure, the insertion of TERC-like sequences was observed only in rodents and not in primates. The only significant difference between the mouse and human TERC structures resides in their 5' ends: while in humans (as well as in many other mammals) the telomeric repeat template lies 45 nucleotides away from the 5' end, in mouse and rat it lies only two nucleotides away [9]. The 43 nucleotide additional sequence appears to play a role in stabilizing the structure of the pseudoknot arm containing the template, maintaining the 5' and the 3' ends of TERC physically close to each other [5]. Therefore, the absence of these 43 nucleotides may allow greater flexibility in the mutual relationship of the 5' and 3' ends of rodent TERC.

The RNA component of telomerase, when inserted with the proposed mechanism, can be considered as a novel transposable element of rodents. Essential functions required for retrotransposition are a reverse transcriptase and an endonuclease coded by the element itself. However, defective elements can be transposed utilizing the required enzymes coded by other transposons (for a review see [62]). In addition, non-long terminal repeat retrotransposons can also be inserted at double-stranded DNA breaks by an endonuclease independent pathway [55,63] and it has been recently shown that, in yeast, RNA can serve as template for the repair of experimentally induced DNA double-strand breaks [64]. Furthermore, some functional relationship between telomerase and endonuclease independendent non-long terminal repeat transposons has emerged [58,65]. The transposition events described here involve a reverse transcriptase (TERT or another reverse transcriptase), coded by a cellular gene, and an RNA (TERC), transcribed from another gene, acting as a transposable element. Thus, the integration of TERC-related fragments can be viewed as endonuclease-independent retrotransposition contributing to the repair of DNA double-strand breaks.

## Conclusion

The data presented here corroborate our hypothesis that the insertion of interstitial telomeric repeats is a consequence of a peculiar pathway of DNA double-strand break repair and extend this conclusion from primates to rodents; we might, therefore, infer that this pathway is more general and probably operates also in other eukaryotes. We also showed that, although rarely, portions of the telomeric RNA other than the canonical template for the telomeric repeats can be retrotranscribed during the process, strongly suggesting the participation of telomerase. These telomerase driven repair processes occurring during evolution constitute a previously undescribed mechanism of genome plasticity and support the hypothesis, based on the structural similarity between telomerase and retrotransposon reverse transcriptases, that an ancient retrotransposon may have provided a DNA-end maintaining activity to the eukaryotic chromosome [65-67].

## Materials and methods

The (TTAGGG)_4 _sequence was used as query for a BLAT search [34] in the genome sequence of the mouse (*M. musculus*: University of California Santa Cruz (UCSC) Genome Browser database, March 2005), rat (*R. norvegicus*: UCSC, June 2003), human (*H. sapiens*: UCSC, July 2003) and chimpanzee (*P. troglodytes*: UCSC, November 2003) [68,69].

A BLAT search of loci containing TERC-like sequences was performed in the genome of the four species using the TERC genes as query [70] (accession numbers: NR_001579, *M. musculus*; NR_001567, *R. norvegicus*; NR_001566, *H. sapiens*; gnl|ti|236061930, *P. troglodytes*). Sequences were aligned using the multiple sequence alignment software, MultAlin [71,72]. The RepeatMasker software [73] was used to identify known repetitive elements.

## Abbreviations

ITS, interstitial telomeric sequence; MYA, million years ago; TERC, telomerase RNA component; TERT, telomerase reverse transcriptase; UCSC, University of California Santa Cruz.

## Authors' contributions

SGN: study conception, research design, data collection, data analysis, manuscript production. MS: data collection, data analysis, manuscript production. AS: manuscript production. CM: data analysis, manuscript production. EG: study conception, research design, data analysis, manuscript writing.

## Additional data files

The following additional data are available with the online version of this paper. Additional data file 1 is a figure summarizing the four mechanisms of ITS insertion previously described [17]. Additional data file 2 comprises two tables: the first table is a list of the 128 mouse loci for which the orthologous rat loci were either not found or grossly rearranged; and the second table is a list of the 120 rat loci not found or rearranged in the mouse genome database. Additional data file 3 lists the 58 ITS loci conserved in the two rodent species. Additional data file 4 comprises two tables in which the mouse-specific and the rat-specific ITSs are listed together with the mechanism of their insertion and the number of nucleotides in register with the inserted telomeric repeats. Additional data file 5 comprises two tables: the first table lists the 75 loci conserved in the two primate species; and the second table reports the three human loci for which the orthologous chimpanzee loci were not found or were grossly rearranged. Additional data file 6 comprises four tables containing the following data sets: **(a) **human-specific ITS loci; **(b) **chimpanzee-specific ITS loci; **(c) **ITS loci inserted before the human-chimpanzee split for which the insertion mechanism was described previously; **(d) **ITS loci conserved in human and chimpanzee and inserted within repetitive elements. Additional data file 7 is a figure reporting the sequence of some examples of species-specific ITS loci and of their ancestral orthologous loci lacking the telomeric repeats. The figure shows how the mechanism of ITS insertion at DNA double strand break sites was deduced. Additional data file 8 is a figure reporting the sequence of all the TERC-ITS loci found in the rodent genomes and a description of their organization.

## Supplementary Material

Additional data file 1 The four mechanisms of ITS insertion previously described [17]. Click here for file

Additional data file 2 The 128 mouse loci for which the orthologous rat loci were either not found or grossly rearranged and the 120 rat loci not found or rearranged in the mouse genome database. Click here for file

Additional data file 3 The 58 ITS loci conserved in the two rodent species. Click here for file

Additional data file 4 Mouse-specific and rat-specific ITSs together with the mechanism of their insertion and the number of nucleotides in register with the inserted telomeric repeats. Click here for file

Additional data file 5 The 75 loci conserved in the two primate species and the three human loci for which the orthologous chimpanzee loci were not found or were grossly rearranged. Click here for file

Additional data file 6 **(a) **Human-specific ITS loci; **(b) **chimpanzee-specific ITS loci; **(c) **ITS loci inserted before the human-chimpanzee split for which the insertion mechanism was described previously; **(d) **ITS loci conserved in human and chimpanzee and inserted within repetitive elements. Click here for file

Additional data file 7 This figure shows how the mechanism of ITS insertion at DNA double strand break sites was deduced. Click here for file

Additional data file 8 The sequence of all the TERC-ITS loci found in the rodent genomes and a description of their organization. Click here for file
