# Supplementary material for: Contribution of telomerase RNA retrotranscription to DNA double-strand break repair during mammalian genome evolution
Source: Genome Biol. 2007 Dec 7;8(12):R260. doi: 10.1186/gb-2007-8-12-r260 (PMC2246262; doi:10.1186/gb-2007-8-12-r260)
Supplement: Additional data file 2 — The 128 mouse loci for which the orthologous rat loci were either not found or grossly rearranged and the 120 rat loci not found or rearranged in the mouse genome database. [file gb-2007-8-12-r260-S2.pdf]

Additional data file 2
Table S1 - Mouse ITS loci for which the orthologous rat loci were not found or were grossly rearranged

	Chromosomal localization	Starting nucleotide of ITS	ITS length (mismatches)
1.	MMU1qA3	19394041	66 (0)
2.	MMU1qA5	22802343	71 (0)
3.	MMU1qC3	69750882	272 (32)
4.	MMU1qE1	100530522	79 (0)
5.	MMU1qF	142388935	29 (1)
6.	MU1qH6	195019658	62 (0)
7.	MU2qA3	19551428	98 (4)
8.	MMU2qB	46157961	62 (0)
9.	MMU2qC1	57555029	239 (3)
10.	MMU2qF1	23517609	69 (3)
11.	MMU3qF2	99742313	130 (10)
12.	MMU4qC5	89701680	61 (0)
13.	MMU4qE2	153937277	35 (1)
14.	MMU5qC2	59650041	56 (0)
15.	MMU5qC3	73581821	83 (5)
16.	MMU5qE5	105238493	84 (1)
17.	MMU5qF	109125203	78 (0)
18.	MMU5qF	120766398	27 (1)
19.	MMU6qB3	53941497	80 (4)
20.	MMU6qB3	56377984	89 (0)
21.	MMU6qC1*	68259674	93 (0)
22.	MMU6qG1	133941357	77 (1)
23.	MMU7qA3	18665269	39 (1)
24.	MMU7qD3	80676175	60 (0)
25.	MMU7qF3	126265763	138 (13)
26.	MMU8qB3	70582624	94 (3)
27.	MMU9qB	54097897	55 (6)
28.	MMU10qA1	5890276	61 (1)
29.	MMU10qA3	22087991	26 (0)
30.	MMU10qB1	37891919	89 (1)
31.	MMU10qB4	62225144	73 (3)
32.	MMU10qB5	72348109	36 (3)
33.	MMU11qA5	40564304	55 (0)
34.	MMU11qB1	57028264	100 (3)
35.	MMU12qD3	88995411	281 (6)
36.	MMU12qF2	110916476	27 (0)
37.	MMU13qA1	14452896	50 (0)
38.	MMU13qA2	18363199	64 (4)
39.	MMU14qE3	104200103	65 (0)
40.	MMU14qE4	107062695	69 (0)
41.	MMU14qE5	118012404	63 (0)
42.	MMU15qF1	100992889	40 (0)
43.	MMU16qA1	3010722	91 (6)
44.	MMU16qC4	95783045	39 (0)
45.	MMU17qA1	10642846	32 (0)
46.	MMU17qA3	29500895	50 (0)
47.	MMU17qC	50686703	156 (4)
48.	MMU17qE5	91027485	73 (1)
49.	MMU17qE5	91628758	67 (1)
50.	MMU18qA1	7076430	81 (0)
51.	MMUXqA1	17995996	54 (0)
52.	MMUXqA6	59154576	40 (2)
53.	MMU1qC1	44497146	65 (1)
54.	MMU1qG1	149394768	28 (0)
55.	MMU1qG1	147608149	74 (5)
56.	MMU1qH4	178494371	67 (1)
57.	MMU2qB	41828439	140 (5)
58.	MMU2qC1	144459165	55 (0)
59.	MMU3qF1	87169949	47 (2)
60.	MMU3qG1	114601741	86 (4)
61.	MMU3qG2	127141085	82 (0)
62.	MMU3qH3	148031423	276 (17)
63.	MMU4qA3	25607856	69 (0)
64.	MMU4qA5	31538583	121 (5)
65.	MMU4qB1	46396183	39 (3)
66.	MMU4qC5	92819479	100 (2)

67.	MMU5qE1	79753757	107 (3)
68.	MMU5qE5	106229832	37 (0)
69.	MMU5qG2	135374985	28 (0)
70.	MMU5qG2	136577105	56 (1)
71.	MMU5qG2	143273417	55 (0)
72.	MMU5qG3	146503825	236 (10)
73.	MMU5qG3	147941306	29 (0)
74.	MMU6qA1	4877085	426 (20)
75.	MMU6qB1	146222214	42 (0)
76.	MMU6qC1	65319212	33 (0)
77.	MMU6qG1	136249298	62 (4)
78.	MMU6qG3	146222214	42 (1)
79.	MMU6qG3	149461913	46 (2)
80.	MMU7qA2	15902452	126 (7)
81.	MMU7qB1	30112225	69 (1)
82.	MMU8qA4	33933969	99 (0)
83.	MMU8qA4	36584734	51 (2)
84.	MMU8qC3	87872719	55 (0)
85.	MMU8qD1	99441586	29 (0)
86.	MMU8qE2	124382305	96 (7)
87.	MMU9qF1	107555844	69 (8)
88.	MMU10qA3*	20386921	108 (4)
89.	MMU10qA4	23884170	79 (5)
90.	MMU10qB4	52395376	64 (0)
91.	MMU10qC1	77766053	80 (6)
92.	MMU10qC2	94566861	107 (2)
93.	MMU10qD1	102582412	77 (1)
94.	MMU10qD1	109282510	37 (0)
95.	MMU10qD3	128822293	31 (0)
96.	MMU11qA5	39050575	46 (0)
97.	MMU11qB1	51208664	259 (20)
98.	MMU11qC*	86742175	55 (6)
99.	MMU11qE2	116247901	24 (0)
100.	MMU12qA1	5539939	41 (0)
101.	MMU12qB1	41720169	30 (0)
102.	MMU12qC1	62475644	37 (0)
103.	MMU12qC3	70620108	66 (0)
104.	MMU12qD1	79134013	60 (3)
105.	MMU12qE	100972302	87 (0)
106.	MMU13qA4	41999177	59 (0)
107.	MMU13qB3	59335002	86 (1)
108.	MMU13qC3	79889500	27 (0)
109.	MMU13qD2	108243674	48 (0)
110.	MMU13qD2	112610858	105 (6)
111.	MMU13qD2	112610858	95 (0)
112.	MMU14qA1	5674431	187 (13)
113.	MMU14qE1	85374410	89 (0)
114.	MMU14qE2	90107006	27 (0)
115.	MMU15qA2	21617684	92 (7)
116.	MMU16qC1	62304223	85 (0)
117.	MMU16qC4	92764017	64 (1)
118.	MMU17qA1	7020493	73 (1)
119.	MMU17qC	48857482	46 (0)
120.	MMU17qE1	60430595	92 (2)
121.	MMU17qE1	63343905	26 (0)
122.	MMU17qE1	64566976	64 (0)
123.	MMU17qE4	85549301	52 (2)
124.	MMU18qE4	88190355	130 (14)
125.	MMU19qD1	49354505	62 (3)
126.	MMU19qD1	50355142	78 (0)
127.	MMUXqE3	122471233	123 (3)
128.	MMUXqF1	134716722	150 (11)

* This ITS is listed in Table 4 as well because it contains a TERC-like fragment

Table S2 - Rat ITS loci for which the orthologous mouse loci were not found or were grossly rearranged

	Chromosomal localization	Starting nucleotide of ITS	ITS length (mismatches)
1.	RNO1p13	164644	88 (3)
2.	RNO1q22	106009847	114 (2)
3.	RNO1q31	131650533	96 (3)
4.	RNO1q33	163818941	76 (4)
5.	RNO1q35	175561089	85 (1)
6.	RNO1q36	183109132	117 (8)
7.	RNO1q41	200487042	61 (1)
8.	RNO1q55	267974943	117 (2)
9.	RNO2q11	1422670	25 (0)
10.	RNO2q11	1683881	77 (1)
11.	RNO2q23	95850058	68 (1)
12.	RNO2q26	127631660	251 (15)
13.	RNO2q26	131484037	46 (1)
14.	RNO2q32	162409549	67 (1)
15.	RNO2q41	209487855	87 (0)
16.	RNO3q11	18944065	71 (0)
17.	RNO3q41	137628190	66 (1)
18.	RNO4q12	16290544	97 (2)
19.	RNO4q22	55452102	141 (14)
20.	RNO4q24	91412687	76 (0)
21.	RNO4q34	113692255	52 (0)
22.	RNO4q42	152782124	62 (0)
23.	RNO5q11	7053449	91 (1)
24.	RNO6q21	58147875	92 (0)
25.	RNO6q32	124711399	77 (1)
26.	RNO6q33	141234118	88 (0)
27.	RNO7q11	293061	39 (1)
28.	RNO7q11	2023033	28 (0)
29.	RNO7q13	32465086	26 (0)
30.	RNO7q22	55554984	183 (10)
31.	RNO7q22	68103256	24 (0)
32.	RNO7q34	113136411	160 (5)
33.	RNO8q11	1030296	97 (4)
34.	RNO8q24	76974148	51 (0)
35.	RNO9q12	9159293	31 (0)
36.	RNO11p11	8555497	68 (0)
37.	RNO11p11	12061065	53 (0)
38.	RNO11q21	48951428	60 (0)
39.	RNO13p13	5179160	98 (4)
40.	RNO13p13	14234017	30 (0)
41.	RNO13p12	20877960	130 (13)
42.	RNO14p22	174304	132 (5)
43.	RNO15p16	6345211	33 (0)
44.	RNO15p14	26976738	273 (13)
45.	RNO15q11	53583323	119 (6)
46.	RNO15q12	65979922	140 (6)
47.	RNO15q21	77130954	435 (4)
48.	RNO15q23	99271717	97 (2)
49.	RNO16p12	33549358	51 (2)
50.	RNO16q12	51992807	82 (1)
51.	RNO16q12	88971954	31 (0)
52.	RNO17p14	1739929	75 (2)
53.	RNO17q12	77356563	74 (0)
54.	RNO18q11	39222133	157 (11)
55.	RNO19p14	640848	98 (0)
56.	RNO19q11	34697675	37 (2)
57.	RNO20p12	280344	74 (0)
58.	RNO20q12	39742133	78 (0)
59.	RNO20q12	41481409	35 (0)
60.	RNOXq21	43855603	93 (5)
61.	RNOXq22	65952063	67 (1)
62.	RNO1p12	20452884	47 (0)
63.	RNO1p11	34478715	62 (1)
64.	RNO1q11	44296656	62 (2)
65.	RNO1q12	53737274	255 (15)
66.	RNO1q22	97292217	63 (0)

67.	RNO1q31	139142856	88 (3)
68.	RNO1q43	208382180	30 (1)
69.	RNO1q41	195142919	87 (2)
70.	RNO1q43	215070728	37 (0)
71.	RNO1q52	233937189	106 (6)
72.	RNO1q53	242560631	128 (6)
73.	RNO1q55	259556514	51 (3)
74.	RNO2q24	113489822	61 (0)
75.	RNO2q41	210756450	83 (1)
76.	RNO2q42	225266594	140 (8)
77.	RNO3q11	21004885	57 (1)
78.	RNO3q42	64252224	26 (0)
79.	RNO4q22	56934688	115 (7)
80.	RNO4q34	118695044	56 (2)
81.	RNO4q42	157332629	34 (0)
82.	RNO4q44	185479314	168 (4)
83.	RNO5q13	30866687	118 (5)
84.	RNO5q22	63676922	26 (0)
85.	RNO5q22	65323533	70 (0)
86.	RNO6q14	25631222	54 (1)
87.	RNO7q31	88176141	52 (1)
88.	RNO7q35	135544846	47 (5)
89.	RNO8q24	61126761	67 (1)
90.	RNO9q11	1565632	69 (0)
91.	RNO9q22	43258238	101 (4)
92.	RNO11q21	64077853	31 (0)
93.	RNO11q23	84836606	61 (0)
94.	RNO12p11	12354519	27 (0)
95.	RNO12q16	34993840	125 (6)
96.	RNO13p13	11099120	24 (0)
97.	RNO13q21	68421549	51 (2)
98.	RNO13q23	82972333	57 (0)
99.	RNO14q11	50824390	27 (0)
100.	RNO14q21	69190935	27 (0)
101.	RNO14q21	93892908	62 (1)
102.	RNO14q22	96532727	81 (0)
103.	RNO15p16	11428279	125 (4)
104.	RNO15q22	87943445	95 (0)
105.	RNO15q23	93088832	130 (6)
106.	RNO15q25	106924507	26 (0)
107.	RNO16p16	4935008	212 (11)
108.	RNO16p13	27007233	75 (0)
109.	RNO16q11	43767568	95 (6)
110.	RNO16q11	51145059	212 (14)
111.	RNO16q12	64930354	63 (0)
112.	RNO16q12	74494133	48 (0)
113.	RNO17p12	41560293	44 (0)
114.	RNO17q12	96417267	117 (3)
115.	RNO18p12	18051078	56 (1)
116.	RNO18q12	80539217	34 (0)
117.	RNO19p11	21760954	25 (0)
118.	RNO20p12	12658773	69 (0)
119.	RNO20p11	24159329	78 (0)
120.	RNOXq21	51133215	88 (2)
