# Supplementary material for: Contribution of telomerase RNA retrotranscription to DNA double-strand break repair during mammalian genome evolution
Source: Genome Biol. 2007 Dec 7;8(12):R260. doi: 10.1186/gb-2007-8-12-r260 (PMC2246262; doi:10.1186/gb-2007-8-12-r260)
Supplement: Additional data file 3 — The 58 ITS loci conserved in the two rodent species. [file gb-2007-8-12-r260-S3.pdf]

Additional data file 3
Table S3 - ITS loci conserved in mouse and rat

	Mouse locus organization			Rat locus organization		
	Chromosomal localization	Starting nucleotide of ITS	ITS length (mismatches)	Chromosomal localization	Starting nucleotide of ITS	ITS length (mismatches)
1.	MMU1qC2	62063616	32 (3)	RNO9q31	60540906	51 (7)
2.	MMU1qC2	64427125	73 (3)	RNO9q32	62951009	22 (0)
3.	MMU1qC3	74158115	135 (19)	RNO9q33	73337466	533 (58)
4.	MMU1qE2	119920380	24 (0)	RNO_rand	57467294	28 (4)
5.	MMU1qE4	131572015	38 (1)	RNO13q13	44660352	199 (10)
6.	MMU2qA1	4433412	59 (0)	RNO17q12	84886191	52 (3)
7.	MMU2qA3	17561003	25 (6)	RNO17q12	91683881	76 (0)
8.	MMU2qE1	91798533	192 (7)	RNO3q24	76382638	64 (3)
9.	MMU2qE3	109539109	17 (3)	RNO3q33	95264034	85 (2)
10.	MMU2qE3	109917404	89 (0)	RNO3q33	95671780	95 (1)
11.	MMU3qF1	85044153	65 (9)	RNO2q34	177050753	80 (1)
12.	MMU4qE2	151645396	581 (35)	RNO5q36	170227213	101 (3)
13.	MMU5qA3	24010932	26 (1)	RNO4q11	4978622	26 (1)
14.	MMU5qB3	45192024	72 (0)	RNO14q21	69805055	164 (18)
15.	MMU5qC3	66308888	25 (2)	RNO14p11	43236526	71 (2)
16.	MMU5qF	112310197	57 (1)	RNO12q16	44270562	62 (0)
17.	MMU5qF	115589154	53 (6)	RNO12q16	41210302	139 (15)
18.	MMU6qB3	51195277	63 (0)	RNO4q24	79591374	159 (8)
19.	MMU6qG3	147451997	68 (3)	RNO4q44	184345247	46 (4)
20.	MMU7qD3	77467335	39 (0)	RNO1q31	139947387	36 (3)
21.	MMU7qF1	108460541	14 (0)	RNO1q34	172785873	94 (4)
22.	MMU8qA2*	21522395	213 (32)	RNO16q12	73726146	58 (8)
23.	MMU8qA2	23608910	35 (2)	RNO16q12	71592478	38 (5)
24.	MMU8qB1	47783105	59 (1)	RNO16q11	46504498	387 (23)
25.	MMU8qD1	95017210	15 (2)	RNO19p13	9793116	43 (1)
26.	MMU8qE1	113750259	68 (1)	RNO19q12	44516093	180 (12)
27.	MMU9qA1	9045604	62 (6)	RNO8q11	5902933	101 (8)
28.	MMU9qA4	27106270	114 (3)	RNO8q13	26818560	42 (4)
29.	MMU9qB	61842383	59 (0)	RNO8q24	65892486	17 (1)
30.	MMU9qE1	82381409	25 (5)	RNO8q31	87181452	147 (11)
31.	MMU9qE3	85594962	15 (2)	RNO8q31	90457904	76 (0)
32.	MMU9qE3	90189609	79 (0)	RNO8q31	95221777	25 (1)
33.	MMU11qA4	32769712	20 (2)	RNO10q12	17751058	26 (0)
34.	MMU12qA1	7171657	77 (3)	RNO6q14	31456143	259 (30)
35.	MMU13qA1	14818453	95 (6)	RNO17q12	57969885	114 (3)
36.	MMU13qA3	30250737	18 (0)	RNO17p12	40169067	109 (0)
37.	MMU14qD1	63964261	139 (26)	RNO15p11	50038098	136 (18)
38.	MMU14qD2	68413206	98 (1)	RNO15q11	54871684	23 (1)
39.	MMU14qE4	113850186	74 (0)	RNO15q24	104335692	26 (1)
40.	MMU15qA1	6105783	79 (3)	RNO2q16	55550830	83 (1)
41.	MMU15qD1	63643128	66 (2)	RNO7q33	100934611	42 (1)
42.	MMU16qA1	5378919	88 (1)	RNO10q12	9970917	96 (11)
43.	MMU16qA1	10868801	22 (0)	RNO10q11	3293349	164 (7)
44.	MMU16qA1	11449519	94 (14)	RNO10q11	2632768	79 (5)
45.	MMU16qA2	15995448	32 (0)	RNO11q23	85816996	19 (2)
46.	MMU16qB3	37593433	136 (14)	RNO11q21	63826479	49 (3)
47.	MMU16qB4	44064610	14 (0)	RNO11q21	57225814	31 (1)
48.	MMU18qE1	62328309	25 (4)	RNO18q12	58166416	55 (0)
49.	MMU18qE2	73371382	42 (3)	RNO18q12	69971922	166 (16)
50.	MMU18qE3	78321060	166 (12)	RNO18q12	75198749	88 (8)
51.	MMU18qE4	85545016	18 (2)	RNO18q12	82103975	46 (0)
52.	MMU19qB	20096670	34 (8)	RNO1q51	224142209	24 (0)
53.	MMU19qC1	26000868	21 (1)	RNO1q51	230330344	33 (1)
54.	MMU19qC1	31269912	144 (7)	RNO1q52	236105371	57 (6)
55.	MMU19qC2	36046186	76 (2)	RNO1q53	241146022	46 (4)
56.	MMUXqA3	33510293	46 (3)	RNOXq11	6336105	137 (6)
57.	MMUXqC3*	94681997	25 (1)	RNO8q31	88164265	43 (4)
58.	MMUXqF2	140480893	30 (4)	RNOXq14	31212745	25 (0)

* Listed also in Table 4
