# Supplementary material for: Contribution of telomerase RNA retrotranscription to DNA double-strand break repair during mammalian genome evolution
Source: Genome Biol. 2007 Dec 7;8(12):R260. doi: 10.1186/gb-2007-8-12-r260 (PMC2246262; doi:10.1186/gb-2007-8-12-r260)
Supplement: Additional data file 4 — Mouse-specific and rat-specific ITSs together with the mechanism of their insertion and the number of nucleotides in register with the inserted telomeric repeats. [file gb-2007-8-12-r260-S4.pdf]

Additional data file 4
Table S4 - Mouse-specific ITS loci

	Mouse locus organization			Rat empty locus organization			Nucleotides in register with the inserted telomeric array	Insertion mechanism
	Chromosomal localization	Starting nucleotide of ITS	Length of telomeric sequence (mismatches)	Chromosomal localization	Starting nucleotide of "empty site"	Length of flanking sequence modification		
1.	MMU1qC3	71374308	74 (0)	RNO9q33	70315182	0	1	No modification
2.	MMU2qE1	91872864	69 (0)	RNO3q24	76463664	0	1	"
3.	MMU3qC3	127866865	101 (4)	RNO2q42	226274249	0	0	"
4.	MMU3qF2	99911261	62 (0)	RNO2q34	195440626	0	2	"
5.	MMU5qG2	130133907	71 (3)	RNO12q12	26586232	0	1	"
6.	MMU9qE3	83888973	55 (1)	RNO8q31	88638371	0	0	"
7.	MMU9qF3	115334624	138 (3)	RNO8q11	119809272	0	6	"
8.	MMU10qA4	30006900	81 (0)	RNO1p11	28586014	0	0	"
9.	MMU12qA1	16114173	100 (1)	RNO6q15	40425216	0	4	"
10.	MMUq12A3	38124518	80 (0)	RNO6q21	60804523	0	3	"
11.	MMU14qD3	80609799	150 (0)	RNO15q12	68344228	0	2	"
12.	MMU15qF3	103164652	47 (2)	RNO7q36	141947229	0	3	"
13.	MMU16qB2	29638411	129 (5)	RNO11q22	71844349	0	4	"
14.	MMU16qB3	33557115	65 (0)	RNO11q22	67908608	0	5	"
15.	MMU18qC	48232284	114 (1)	RNO18q11	42680339	0	1	"
16.	MMU2qC1	66044880	274 (17)	RNO3q21	48046795	5	5	Deletion
17.	MMU2qH3	165898759	174 (16)	RNO3_Rand	1442317	8	5	"
18.	MMU2qH4	180305852	27 (0)	RNOq43	169585253	14	0	"
19.	MMU3qD	59598683	60 (0)	RNO2q31	149152710	55	0	"
20.	MMU3qF2	102874350	57 (1)	RNO2q34	198648736	16	1	"
21.	MMU3qH4	158927773	28 (0)	RNO2q45	258112731	5	1	"
22.	MMU4qC6	98356530	76 (1)	RNO5q33	120026192	4	1	"
23.	MMU5qE3	92625465	77 (0)	RNO14p22	16323799	17	3	"
24.	MMU7qA1	13485079	30 (0)	RNO1q21	76873665	5	1	"
25.	MMU7qF4	132979741	78 (2)	RNO1q41	198553718	5	2	"
26.	MMU8qA1	17219445	49 (0)	RNO16q12	77268741	6	3	"
27.	MMU9qE3	95334838	132 (5)	RNO8q31	100534657	10	4	"
28.	MMU10qA3	18627223	49 (3)	RNO1p12	13804580	84	0	"
29.	MMU10qC2	94845948	75 (0)	RNO7q13	32086310	17	8	"
30.	MMU10qD2	123367729	48 (0)	RNO7q22	63491942	16	0	"
31.	MMU11qA5	40477183	43 (0)	RNO10q12	25767746	5	1	"
32.	MMU11qB3	64408301	104 (7)	RNO10q24	51014457	7	2	"
33.	MMU13qC3	90125363	88 (5)	RNO2p12	24144489	13	3	"
34.	MMU14qA1	10994375	67 (1)	RNO16p16	14152855	4	0	"
35.	MMU14qC2	53194069	109 (3)	RNO15p12	37725128	8	2	"
36.	MMU15qE2	85590832	61 (0)	RNO7q34	123572670	5	3	"
37.	MMU15qE3	88020385	121 (2)	RNO7q34	126305612	32	2	"
38.	MMU18qB3	43134545	97 (3)	RNO18p11	36195781	6	0	"
39.	MMU19qC1	31632160	54 (0)	RNO1q52	236482320	45	2	"
40.	MMU19qD2	58082329	49 (0)	RNO1q55	265111070	9	1	"
41.	MMUXqA1	12978918	111 (8)	RNOXq12	20029300	7	0	"
42.	MMUXqE3	127695000	290 (24)	RNOXq34	121683365	34	2	"
43.	MMU1qH6	192966577	112 (0)	RNO13q27	109265521	2	NI	Random Seq Add
44.	MMU1qH6	192192893	91 (0)	RNO13q27	108546028	5	NI	"
45.	MMU2qH4	178503999	54 (0)	RNO3q43	167805605	39	2	"
46.	MMU3qG1	120587839	63 (4)	RNO2q41	218407148	6	NI	"
47.	MMU8qE2	123248677	122 (7)	RNO19q12	54165535	141	NI	"
48.	MMU9qF3	113313368	63 (2)	RNO8q32	117921021	1	0	"
49.	MMU18qA2	23040469	62 (4)	RNO18p12	14490884	2	NI	"
50.	MMU18qE4	89175564	51 (0)	RNO18q13	85967271	3	2	"
51.	MMU3qA3	21038765	89 (0)	RNO2q24	107072450	11	3	Duplication
52.	MMU12qE	98339162	62 (0)	RNO6q32	127158889	18	3	"
53.	MMU11qE2	120349304	53 (0)	RNO10q32	109932417	46/91	NI	Random Seq Add/Del
54.	MMU1qC3*	69326366	139 (16)	RNO9q32	68032617	50	NI	TERC Add
55.	MMU9qA5*	47975239	66 (0)	RNO8q23	51245798	65	NI	"
56.	MMU1qC1*	47024585	57 (0)	RNO9q22	52312295	31/126	NI	TERC Add/Del

57.	MMU4qD2*	119006631	53 (3)	RNO5q36	140562692	42/11	NI	“
58.	MMU10qB4*	58505217	27 (1)	RNO20q11	37230663	118/7	NI	“

* Listed also in Table 4
NI, Not informative
Seq, sequence
Add, addition
Del, deletion

Table S5 - Rat-specific ITS loci

	Rat locus organization			Mouse empty locus organization			Nucleotides in register with the inserted telomeric array	Insertion mechanism
	Chromosomal localization	Starting nucleotide of ITS	Length of telomeric sequence (mismatches)	Chromosomal localization	Starting nucleotide of “empty site”	Length of flanking sequence modification		
1.	RNO1q33	170404611	60 (0)	MMU7qE3	106338358	0	1	No modification
2.	RNO2q44	244500676	45 (4)	MMU3qH2	145265356	0	0	“
3.	RNO3q36	115152268	58 (0)	MMU2qF1	127237595	0	6	“
4.	RNO3q43	170542669	32 (0)	MMU2qH4	181218602	0	2	“
5.	RNO4q33	106635857	70 (0)	MMU6qC1	73387975	0	4	“
6.	RNO6q24	93355784	96 (2)	MMU12qC2	68091145	0	5	“
7.	RNO7q34	109642996	61 (0)	MMU15qD3	71925048	0	1	“
8.	RNO7q34	116325182	76 (2)	MMU15qE1	78421075	0	2	“
9.	RNO10q26	74713422	63 (0)	MMU11qC	86184449	0	3	“
10.	RNO13q22	77344646	53 (0)	MMU1qH2	161559860	0	1	“
11.	RNO14p11	33782205	30 (0)	MMU5qC3	75910183	0	0	“
12.	RNO14q21	78916865	106 (3)	MMU5qB3	35932974	0	3	“
13.	RNO17p12	37360246	73 (0)	MMU13qA3	33425385	0	2	“
14.	RNO17q12	55663586	70 (1)	MMU13qA2	17018301	0	1	“
15.	RNO18q11	47451682	118 (2)	MMU18qD1	52292258	0	0	“
16.	RNOXq14	38375105	57 (0)	MMUXqF3	146739252	0	0	“
17.	RNO1q32	158508662	94 (0)	MMU7qE1	95094008	I2	0	Deletion
18.	RNO1q41	191523325	75 (2)	MMU7qF3	126149266	2	1	“
19.	RNO1q55	264659404	98 (1)	MMU19qD2	57679643	4	0	“
20.	RNO3q35	101101050	28 (0)	MMU2qE4	114746079	8	1	“
21.	RNO4q44	176770103	62 (0)	MMU6qG2	140196015	25	1	“
22.	RNO5q31	102846995	57 (1)	MMU4qC3	82992642	22	2	“
23.	RNO5q36	140423491	110 (3)	MMU4qD2	118860955	68	4	“
24.	RNO5q36	170737787	66 (0)	MMU4qE2	152431963	5	3	“
25.	RNO6q31	115393976	62 (1)	MMU12qD3	87181783	9	0	“
26.	RNO7q36	142292316	71 (0)	MMU15qF3	103364972	2	3	“
27.	RNO8q32	123589071	118 (6)	MMU9qF3	118646308	14	2	“
28.	RNO9q31	53165600	52 (0)	MMU1qC1	54667318	2	3	“
29.	RNO10q22	37198112	135 (5)	MMU11qB1	51430914	44	1	“
30.	RNO10q24	52336121	51 (0)	MMU11qB3	65791231	10	4	“
31.	RNO11q23	83310408	60 (0)	MMU16qA3	18724482	6	3	“
32.	RNO12q11	15821984	40 (0)	MMU5qG2	138355030	86	3	“
33.	RNO14p22	2474415	31 (0)	MMU5qF	106897508	17	4	“
34.	RNO14q22	106891412	34 (0)	MMU11qA3	25698816	10	3	“
35.	RNO15p16	2025994	57 (0)	MMU14qA3	20659475	1	2	“
36.	RNO15p16	13792982	72 (0)	MMU14qA1	11352844	16	3	“
37.	RNO15p11	51765075	79 (3)	MMU14qD1	65633532	3	1	“
38.	RNO15q25	109409344	96 (0)	MMU14qE5	118836002	10	5	“
39.	RNO16p16	2456167	78 (0)	MMU14qA3	25329537	19	4	“
40.	RNO16p15	11869377	106 (4)	MMU14qB	34116313	11	0	“
41.	RNO16p12	31850136	48 (1)	MMU8qB3	60660440	42	0	“
42.	RNO16q12	55014499	63 (0)	MMU8qA4	39547909	49	1	“
43.	RNO16q12	65187785	62 (0)	MMU8qA2	29662906	2	3	“
44.	RNO16q12	89633150	25 (0)	MMU8qA1	4775109	6	2	“
45.	RNO17p14	3885471	87 (0)	MMU13qB3	65599346	17	1	“
46.	RNO18p12	25216364	36 (1)	MMU18qB1	32959779	7	1	“
47.	RNO19p11	16792421	35 (0)	MMU8qC4	90669596	2	0	“
48.	RNO19q11	32022918	88 (0)	MMU8qC1	76960105	6	5	“
49.	RNO19q12	55864771	30 (0)	MMU8qE2	124937599	11	1	“
50.	RNO20p11	26445860	81 (0)	MMU10qB5	63952561	1107	2	“
51.	RNO20q11	36173787	88 (3)	MMU10qB4	57348931	2	2	“
52.	RNOXq12	15324067	101 (3)	MMUXqA1	17126359	7	0	“
53.	RNOXq13	24504883	59 (0)	MMUXqA1	8732733	10	4	“
54.	RNOXq37	159499984	63 (0)	MMUXqA7	68187963	5	4	“
55.	RNO5q36	163728884	24 (0)	MMU4qE1	144184636	8	NI	Random Seq Add
56.	RNO6q23	76591610	93 (7)	MMU12qC1	52913469	1	NI	“
57.	RNO7q33	100554100	147 (6)	MMU15qD1	63366082	29	NI	“

58.	RNO8q31	97024675	43 (0)	MMU9qE3	91919459	24	1	“
59.	RNO9q11	1630245	77 (0)	MMU17qC	50937699	8	NI	“
60.	RNO11q11	21546182	32 (0)	MMU16qC3	81302928	15	0	“
61.	RNO14q11	64540088	61 (1)	MMU5qC1	50101946	6	1	“
62.	RNO14q11	66506534	89 (0)	MMU5qB3	48072607	226	NI	“
63.	RNO14q21	89089267	71 (3)	MMU11qA1	8397275	3	NI	“
64.	RNO16p14	23309378	29 (0)	MMU8qB3	67168858	32	0	“
65.	RNOXq31	92082288	218 (4)	MMUXqD	98607768	50	NI	“
66.	RNOXq36	146334120	110 (3)	MMUXqA6	55332956	34	0	“
67.	RNO1q32	159960687	116 (12)	MMU7qE2	96491722	14	0	Duplication
68.	RNO2q34	181871056	69 (0)	MMU3qF1	89474328	5	1	“
69.	RNO9q33	70280330	98 (4)	MMU1qC3	71337574	187	2	“
70.	RNOXq21	45438697	127 (5)	MMUXqF5	163069120	12	0	“
71.	RNO7q21	44474538	181 (13)	MMU10qD1	105687267	19/4	NI	Random Seq Add/Del
72.	RNO18p13	3939241	87 (1)	MMU18qA1	12604587	39/60	NI	“

NI, Not informative
Seq, sequence
Add, addition
Del, deletion
