# Supplementary material for: Contribution of telomerase RNA retrotranscription to DNA double-strand break repair during mammalian genome evolution
Source: Genome Biol. 2007 Dec 7;8(12):R260. doi: 10.1186/gb-2007-8-12-r260 (PMC2246262; doi:10.1186/gb-2007-8-12-r260)
Supplement: Additional data file 5 — The 75 loci conserved in the two primate species and the three human loci for which the orthologous chimpanzee loci were not found or were grossly rearranged. [file gb-2007-8-12-r260-S5.pdf]

Additional data file 5
Table S6 - ITS loci conserved in human and chimpanzee

	Human locus organization			Chimpanzee locus organization		
	Chromosomal localization	Starting nucleotide of ITS	ITS length (mismatches)	Chromosomal localization	Starting nucleotide of ITS	ITS length (mismatches)
1.	HSA1p36	11209776	64 (3)	PTR1	11646639	96 (8)
2.	HSA1p36	18013967	34 (0)	PTR1	18533223	22 (0)
3.	HSA1p34	41160952	44 (2)	PTR1	42378933	38 (2)*
4.	HSA1p13	78017054	36 (0)	PTR1	76901942	36 (0)
5.	HSA2p12	75274210	42 (4)	PTR12	77616525	42 (4)
6.	HSA2q14	123012039	38 (3)	PTR13	8662344	38 (3)
7.	HSA2q31	182343128	69 (1)	PTR13	71716824	57 (1)*
8.	HSA2q36	223130670	50 (4)	PTR13	113326474	38 (4)
9.	HSA2q36	230182006	27 (2)	PTR13	120433530	27 (2)
10.	HSA2q37	233522426	25 (1)	PTR13	123736397	37 (2)*
11.	HSA2q37	241482028	35 (0)	PTR13	35180448	88 (2)*
12.	HSA3p14	59364839	25 (0)	PTR2	61115780	25 (0)
13.	HSA4p16	6490170	39 (2)	PTR3	6607468	39 (2)
14.	HSA4p16	8541680	45 (3)	PTR3	8544082	39 (3)
15.	HSA4p12	47122044	27 (1)	PTR3	99870955	27 (0)
16.	HSA5p15	16400930	41 (2)	PTR4	16829183	41 (0)
17.	HSA5p13	40774567	42 (3)	PTR4	75782701	42 (5)
18.	HSA5q14	82896052	41 (2)	PTR4	32318230	23 (0)*
19.	HSA6p24	1060028	39 (1)	PTR5	1068621	39 (1)*
20.	HSA6p21	38959659	78 (4)	PTR5	39570154	60 (3)
21.	HSA6q22	135856130	29 (1)	PTR5	138701406	29 (1)
22.	HSA7p22	972655	33 (1)	PTR6	1060973	27 (2)
23.	HSA7q36	151986856	53 (0)	PTR6	17600852	41 (0)*
24.	HSA7q36	154981811	50 (3)	PTR6	157876908	62 (3)*
25.	HSA7q36	155395578	28 (0)	PTR6	158284654	28 (0)
26.	HSA8p12	35523062	39 (1)	PTR7	36796288	39 (1)*
27.	HSA8p12	37151482	28 (1)	PTR7	38433956	28 (2)
28.	HSA8q21	76951504	26 (1)	PTR7	79138810	26 (1)
29.	HSA9p24	2814045	37 (0)	PTR11	2778776	25 (0)*
30.	HSA9q21	67337343	71 (3)	PTR11	52886831	47 (2)
31.	HSA9q34	128303776	100 (11)	PTR11	114929189	94 (9)
32.	HSA9q34	132545184	33 (1)	PTR11	119195874	33 (2)
33.	HSA9q34	136228252	25 (2)	PTR11	122972062	37 (3)
34.	HSA10p15	3940375	190 (28)	PTR8	4039317	309 (47)
35.	HSA10p15	4065429	125 (18)	PTR8	4163428	142 15)
36.	HSA10p14	8514520	159 (13)	PTR8	8653542	108 12)
37.	HSA10p11	46982375	33 (1)	PTR8	30811377	33 (2)
38.	HSA10q22	79979595	52 (7)	PTR8	81693960	53 (6)
39.	HSA11p15	1442570	24 (2)	PTR9	1570832	24 (0)
40.	HSA11p15	2109783	25 (0)	PTR9	2298761	25 (0)
41.	HSA11p15	10651009	27 (0)	PTR9	10879728	19 (1)
42.	HSA11p14	25512080	24 (0)	PTR9	25818465	24 (0)
43.	HSA11q24	123941136	37 (2)	PTR9	125987562	37 (2)
44.	HSA12p13	87553	35 (0)	PTR10	105275	29 (0)*
45.	HSA12p11	22285277	35 (1)	PTR10	22919421	35 (1)*
46.	HSA12q24	125403098	74 (7)	PTR10	128685625	74 (7)
47.	HSA13q31	80079215	25 (1)	PTR14	63870240	27 (1)
48.	HSA13q31	92642532	21 (0)	PTR14	76770966	27 (0)
49.	HSA13q34	110601947	164 (26)	PTR14	95321714	158 (27)
50.	HSA14q32	97833450	27 (2)	PTR15	99410725	33 (2)
51.	HSA15q11	18650515	19 (0)	PTR16	28031674	24 (0)
52.	HSA15q22	69007769	28 (2)	PTR16	69709477	28 (2)*
53.	HSA15q25	78471632	31 (0)	PTR16	79254569	25 (0)
54.	HSA16p13	65241	16 (0)	PTR18	68110	34 (0)
55.	HSA16p13	10314218	31 (1)	PTR18	10388919	37 (2)
56.	HSA16p12	25738028	55 (1)	PTR18	26358917	61 (1)
57.	HSA16q22	65053274	49 (5)	PTR18	58050751	47 (4)

58.	HSA16q23	75930570	55 (6)	PTR18	69173216	50 (5)
59.	HSA16q24	88690816	37 (1)	PTR18	82476983	53 (2)
60.	HSA17p12	12576423	25 (2)	PTR19	26052515	25 (2)
61.	HSA17q25	78735795	21 (0)	PTR19	79337810	27 (0)
62.	HSA18q21	59791805	25 (1)	PTR17	56645827	25 (1)
63.	HSA19q13	21981095	38 (4)	PTR20	22928526	38 (5)*
64.	HSA20p12	10167184	34 (1)	PTR21	9984464	34 (2)*
65.	HSA21q21	22056873	42 (4)	PTR22	22224323	42 (4)
66.	HSA21q22	35005625	84 (6)	PTR22	35002687	66 (5)
67.	HSA21q22	38128537	35 (0)	PTR22	38168382	51 (0)
68.	HSA21q22	44708085	75 (7)	PTR22	44847640	75 (7)
69.	HSA22q13	43298817	52 (2)	PTR23	43656317	32 (1)
70.	HSA22q13	41492788	31 (0)	PTR23	41845876	31 (0)
71.	HSAXp22	2888475	57 (6)	PTRX	3051845	57 (6)
72.	HSAXp22	5490581	34 (2)	PTRX	5855638	34 (2)
73.	HSAXq28	153138584	35 (1)	PTRX	159591531	35 (1)
74.	HSAY	6832085	42 (3)	PTRY	7083137	36 (3)*
75.	HASY	12475024	52 (4)	PTRY	12939534	52 (4)

* ITS inserted in repetitive element, insertion mechanism was defined from repetitive element consensus and included in Table 1 and in Additional data file 6

Table S7 - Human ITS loci for which the orthologous chimpanzee loci were not found or were grossly rearranged

	Chromosomal localization	Starting nucleotide of ITS	ITS length (mismatches)
1.	HSA11q1	62571027	55 (3)
2.	HSAY	18254397	26 (1)
3.	HSAXp11	49665897	55 (4)
