# Supplementary material for: Contribution of telomerase RNA retrotranscription to DNA double-strand break repair during mammalian genome evolution
Source: Genome Biol. 2007 Dec 7;8(12):R260. doi: 10.1186/gb-2007-8-12-r260 (PMC2246262; doi:10.1186/gb-2007-8-12-r260)
Supplement: Additional data file 6 — (a) Human-specific ITS loci; (b) chimpanzee-specific ITS loci; (c) ITS loci inserted before the human-chimpanzee split for which the insertion mechanism was described previously; (d) ITS loci conserved in human and chimpanzee and inserted within repetitive elements. [file gb-2007-8-12-r260-S6.pdf]

Additional data file 6
Table S8 - Human and chimpanzee ITS loci with a defined mechanism of insertion

A. Human-specific ITS loci

	Human locus localization			Chimpanzee empty locus organization		Length of flanking sequence modification	Nucleotides in register with the inserted telomeric array	Insertion mechanism	Ref.
	Chromosomal localization	Starting nucleotide of ITS	Length of telomeric sequence (mismatches)	Chromosomal localization	Starting nucleotide of “empty site”				
1.	HSA15q14	39959290	34 (0)	PTR16	40004170	0	0	No modification	a
2.	HSA3q25	160732967	41 (1)	PTR2	164068022	1	3	Deletion	a
3.	HSA16q22	75147692	41 (0)	PTR18	68324975	5	4	“	a
4.	HSA11q24	129111094	32 (1)	PTR9	131211358	7	2	Random seq add	b
5.	HSA9q34	126292595	55 (0)	PTR11	112827938	15	1	Duplication	b

B. Chimpanzee-specific ITS loci

	Chimpanzee locus localization			Human empty locus organization		Length of flanking sequence modification	Nucleotides in register with the inserted telomeric array	Insertion mechanism	Ref.
	Chromosomal localization	Starting nucleotide of ITS	Length of telomeric sequence (mismatches)	Chromosomal localization	Starting nucleotide of “empty site”				
1.	PTR13	128434905	34 (0)	HSA2q37	237999141	15	2	Deletion	b
2.	PTR22	41998107	24 (0)	HSA21q22	41943110	7	3	"	b
3.	PTR22	18816173	43 (0)	HSA21q21	18699305	76	?	Random seq add	a
4.	PTR12	85987285	35 (0)	HSA2p12	83180613	15	0	Duplication	b

C. ITS loci inserted before the human-chimpanzee split

	Human locus localization			Chimpanzee locus localization			Length of flanking sequence modification	Nucleotides in register with inserted telomeric array	Insertion mechanism	Ref.
	Chromosomal localization	Starting nucleotide of ITS	Length of telomeric sequence (mismatches)	Chromosomal localization	Starting nucleotide of ITS	Length of telomeric sequence (mismatches)				
1.	HSA6p24	1060028	39 (1)	PTR5	1068621	39 (1)	0	2	No modification	a
2.	HSA7q36	151986856	53 (0)	PTR6	17600852	41 (0)	18	3	Deletion	a
3.	HSA8p12	35523062	39 (1)	PTR7	36796288	39 (1)	23	3	"	a
4.	HSA9p24	2814045	37 (0)	PTR11	2778776	25 (0)	42	0	"	a
5.	HSA7q36	154981811	50 (3)	PTR6	157876908	62 (3)	8	?	Random seq add	a
6.	HSA2q31	182343128	69 (1)	PTR13	71716824	57 (1)	43	2	Duplication	a
7.	HSA5q14	82896052	41 (2)	PTR4	32318230	23 (0)	15	15	"	a

D. ITS loci conserved in human and chimpanzee and inserted within repetitive elements

	Human locus localization			Chimpanzee locus localization			Repetitive element organization			Insertion mechanism	Ref.
	Chromosomal localization	Starting nucleotide of ITS	Length of telomeric sequence (mismatches)	Chromosomal localization	Starting nucleotide of ITS	Length of telomeric sequence (mismatches)	Interrupted repetitive element (insertion breakpoint)	Length of flanking sequence modification	Nucleotides in register with inserted telomeric array		
1.	HSA2q37	233522426	25 (1)	PTR13	123736397	37 (2)	L1MB2#LINE/L1 (753)	0	3	No modification	b
2.	HSAY	6832085	42 (3)	PTRY	7083137	36 (3)	MER41B#LTR/ERV1 (349)	0	0	"	b
3.	HSA1p34	41160952	44 (2)	PTR1	42378933	38 (2)	MIRb#SINE/MIR (41)	2	0	Deletion	b
4.	HSA2q37	241482028	35 (0)	PTR13	35180448	88 (2)	L1M4_orf2#LINE/L1 (1532)	10	0	"	b
5.	HSA12p13	87553	35 (0)	PTR10	105275	29 (0)	HERVP71A#LTR/ER (4270)	6	1	"	b
6.	HSA12p11	22285277	35 (1)	PTR10	22919421	35 (1)	AluJb#SINE/Alu (223)	6	0	"	b
7.	HSA15q22	69007769	28 (2)	PTR16	69709477	28 (2)	L1M4_5end#LINE/L1 (687)	13	4	"	b
8.	HSA20p12	10167184	34 (1)	PTR21	9984464	34 (2)	L2#LINE/L2 (2584)	1	0	"	b
9.	HSA19q13	21981095	38 (4)	PTR20	22928526	38 (5)	HERV70#LTR/ERV1 (2077)	7	0	Random seq add	b

a Nergadze et al., 2004
b This paper
